# Differential substrate preferences IN ACTINOBACTERIAL protein *O*-MANNOSYLTRANSFERASES and alteration of protein-*O*-MANNOSYLATION by choice of secretion pathway

**DOI:** 10.1093/glycob/cwae095

**Published:** 2024-12-03

**Authors:** Hirak Saxena, Rucha Patel, John Kelly, Warren Wakarchuk

**Affiliations:** Department of Biological Sciences, University of Alberta, 116 St & 85 Ave, Edmonton, AB T6G 2R3; Department of Biological Sciences, University of Alberta, 116 St & 85 Ave, Edmonton, AB T6G 2R3; Human Health Therapeutics, National Research Council of Canada, 100 Sussex Dr, Ottawa, ON K1N 1J1; Department of Biological Sciences, University of Alberta, 116 St & 85 Ave, Edmonton, AB T6G 2R3

**Keywords:** Actinobacteria, Protein-*o*-mannosylation, Protein-*o*-mannosyltransferase

## Abstract

Protein-*O*-mannosylation (POM) is a form of *O*-glycosylation that is ubiquitous and has been studied extensively throughout in fungi and animals. The key glycosyltransferase, protein *O*-mannosyltransferase (PMT), a member of family GT-39, is also found in over 3,800 bacterial genomes but has only been minimally examined from prokaryotes. In prokaryotes POM has only been investigated in terms of pathogenicity (in *Mycobacterium tuberculosis*) even though there are far more non-pathogenic bacteria that appear to carry out POM. To date, there is no consensus on what benefit POM imparts to the non-pathogenic bacteria that can perform it. Through the generation of a POM deficient mutant of *Corynebacterium glutamicum* – a widely utilized and known protein *O*-mannosylating actinobacteria – this work shows that even closely related actinobacterial GT-39 s (the enzymes responsible for the initiation of POM) can have different substrate specificities for targets of POM. Moreover, presented here is evidence that POM does not only occur in a SEC-dependent manner; POM also occurs with TAT and non-SEC secreted substrates in a specific and likely tightly regulated manner. Together these results highlight the need for further biochemical characterization of POM in these and other bacterial species to help elucidate the true nature of its biological functions.

## Introduction

POM is an essential and ubiquitous posttranslational modification found in fungi and animals (Lommel and Strahl 2009). It has also been described in some actinobacteria (Béguin and Eisen 1978; Wakarchuk et al. 2016; Howlett et al. 2018; Keenan et al. 2019) including *M. tuberculosis* where POM was demonstrated to play a critical role in the virulence (C. F. Liu et al. 2013), but no definitive biological context has been attributed to it in the non-pathogenic members of the phylum highlighting a significant lack in the understanding of prokaryotic POM. Most of the detailed information on POM and the PMT enzymes responsible for it has been garnered from work done in *Saccharomyces cerevisiae*, which has contributed significantly to the understanding of this process in eukaryotes (Loibl and Strahl 2013; Bai et al. 2019). To date only a few of the mannosylated proteins identified in well-known actinobacteria like *M. tuberculosis* (Espitia and Mancilla 1989; VanderVen et al. 2005; González-Zamorano et al. 2009), *C. glutamicum* (Hartmann et al. 2004; Mahne et al. 2006), *Cellulomonas fimi* (Langsford et al. 1984, 1987), and *Streptomyces coelicolour* (Wehmeier et al. 2008; Keenan et al. 2019) have had functions attributed to them, complicating the elucidation of the biological context of this modification. This is despite there being over 3800 bacterial species with an annotated PMT – belonging to glycosyltransferase family 39 (GT-39) in the Carbohydrate Active Enzyme (CAZy) database (Drula et al. 2022).

Previously, POM was thought to occur in a SEC dependant manner during extracellular translocation across the plasma membrane (VanderVen et al. 2005); however, many TAT-exported proteins have also been identified to be O-mannosylated, which are folded prior to their export (Posey et al. 2006; Xu et al. 2013; Keenan et al. 2019). Recently, mannosylated cytosolic and non-SEC translocon secreted proteins have also been identified in *C. fimi* and *Cellulomonas flavigena* (Wakarchuk et al. 2016; Saxena et al. 2023). This contradiction serves to highlight the fact that there is still a significant lack of information on both the mechanisms involved in bacterial protein-*O*-mannosylation and its overall function for the bacterium.

There are four generalized steps to protein-O-mannosylation: firstly, a mannosylated lipid donor is synthesized through the action of a GT-2 family glycosyltransferase (known as Ppm1 in actinobacteria), that transfers mannose from the activated donor GDP-α-D-mannose to the phosphorylated prenyl or dolichol donor lipid. Next, the phosphomannose-lipid is flipped across the membrane by a still unknown protein to where it can interact with the PMT, which then transfers the mannose through an inverting mechanism to the hydroxyl group of Ser or Thr residues on the target protein as it is translocated across a biological membrane (Tanner 1969; Orlean et al. 1988; Girrbach et al. 2000; Kruszewska et al. 2000; Willer et al. 2003; Maeda and Kinoshita 2008). The mono-mannosylated glycoprotein will then undergo further modification by distinct enzymes to complete the glycan, producing the final glycoprotein product (Strahl-Bolsinger et al. 1999). While the enzymes responsible for the subsequent elongation of mannoglycans in actinobacteria is not currently known, it is likely performed by GT-4 family members recognized to have α-1,2-, α-1,3-, and/or α-1,6-mannosyltransferase activity (7 of which are annotated in the *C. glutamicum* ATCC 13032 genome).

All PMT enzymes identified to date are integral membrane proteins containing several transmembrane domains and contain enough sequence homology to be grouped into GT-39, highlighting the conservation of *O*-mannoslyation (Drula et al. 2022).. Recently, the first cryo-electron microscopy structure of a GT-39 PMT1-PMT2 heterodimer from *S. cerevisiae* was solved, while only topology reports and hydropathy profiles of several bacterial PMTs have been published (Lommel et al. 2011; Vigerust 2011; Bai et al. 2019).

Across species, GT-39 s can show a marked degree of variation. The only prokaryotic GT-39 s investigated to date originated from *M. tuberculosis* and *C. glutamicum* and show only 25.7% and 26% global sequence similarity, respectively, to the PMT1 of *S. cerevisiae*. Interestingly, these two prokaryotic PMTs share only a 55.9% similarity (Table S1), which could be partially explained by their taxonomic relation. Genomic analyses have revealed other putative bacterial GT-39 s in other actinobacteria, like *C. fimi* and *C. flavigena* (25.4% and 23.2% similarity to *S. cerevisiae* PMT1, 46% and 46.9% similarity to the *M. tuberculosis* GT-39, and 42% and 40.5% similarity to the *C. glutamicum* GT-39, respectively).

*C. glutamicum* is a Gram-positive non-pathogenic, non-sporulating, non-motile rod-shaped bacteria that has been widely utilized in industrial applications such as the production of amino acids, nucleotides, and enzymes (Mori and Cline 2001; Sanli 2004; Tsuchidate et al. 2011). *C. glutamicum*, *M. tuberculosis*, *C. fimi*, and *C. flavigena* are all members of the Actinomycetales order and have genomes that are high in G + C% content. The wide utilization of *C. glutamicum* means there exists an established molecular toolbox for genetic engineering of this organism. In addition, the close genetic resemblance of *C. glutamicum* to the previously mentioned actinobacteria (and common mannosylation machinery) suggests that this organism is an ideal candidate to serve as a Gram-positive protein expression/secretion system for the purpose of investigating actinobacterial POM. This was demonstrated recently, as we showed the *C. glutamicum* platform was shown to accurately *O*-mannosylate and localize recombinantly produced heterologous proteins originating from *C. fimi* (Saxena et al. 2023).

A GT-39 deficient mutant of *C. glutamicum* recombinantly producing actinobacterial PMTs (from *C. glutamicum*, *C. fimi*, and *C. flavigena*) will allow for the in vivo assay of these integral, multipass transmembrane proteins, which are traditionally difficult to biochemically characterize and therefore under reported in the literature. Utilizing these complemented strains to produce actinobacterial mannoproteins from each species will show any potential differences in substrate preference between the related actinobacterial GT-39 s, allowing for the continued development of the *C. glutamicum* platform for the investigation of POM in actinobacteria.

## Results

### Cg_1014 knockout and complementation

**Fig. 1:**
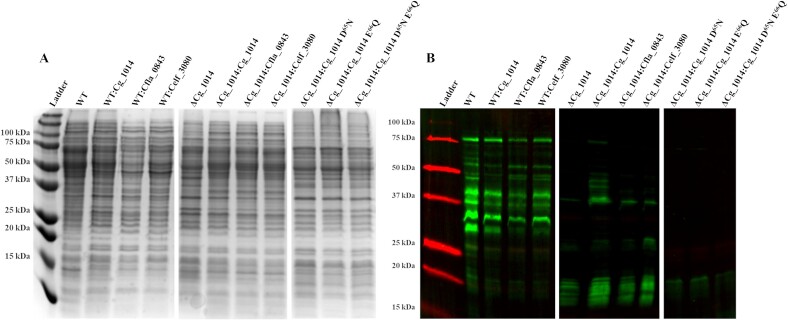
**Coomassie stained 15% SDS-PAGE (A) and ConA-FITC lectin blot (B) of *C. Glutamicum* ATCC 13032 and ΔCg_1014 membrane fractions expressing recombinant actinobacterial GT-39 s and Cg_1014 SDM constructs.** Coomassie stained gel shown for total loading (A). Overexpression of the actinobacterial GT-39 s in *C. Glutamicum* ATCC 13032 resulted in no changes to the native membrane protein mannoproteome as detected by Concanavalin A-FITC lectin conjugate (B). Western blotting using anti-HIS_6_ antibodies and fluorescent Ni-NTA conjugates did not detect any proteins indicative of recombinant GT-39 s. lack of POM in the ΔCg_1014 strain was partially complemented by expression of the *C. Glutamicum* GT-39 Cg_1014, with no complementation by the *C. Fimi* or *C. Flavigena* GT-39 s (Celf_3080 and Cfla_0843, respectively). Substitution of the conserved residues D^65^ and E^66^ to N^65^ and Q^66^, respectively, confirmed their requirement for catalytic activity. Molecular weight standards are the bio-rad all blue ladder.

To adequately assess in vivo POM by each recombinantly expressed actinobacterial GT-39, the native *C. glutamicum* GT-39 was inactivated. Homologous recombination was used to knockout the PMT gene in *C. glutamicum* (Cg_1014), taking advantage of the wide host range of the pK18mobsacB (Schäfer et al. 1994) suicide vector. Predictive software tools revealed the possibility of regulatory transcriptional elements for neighbouring genes to be contained on the non-coding strand of Cg_1014, specifically, the regions that coded for the N- and C- termini of the PMT enzyme. For this reason, a truncated and inactive knockout construct was designed instead of a seamless knockout (Fig. S1). This truncated construct only consisted of the cytoplasmic N-terminal region, the first transmembrane region, and the extracellular C-terminal region. As the putative active site and highly conserved residues D^65^ and E^66^ are contained in the first extracellular loop, it was predicted that this construct would effectively abolish POM in *C. glutamicum*. Following homologous recombination, clones were screened and confirmed via colony PCR (Fig. S2) for replacement of the native Cg_1014 gene by the truncated and inactive knockout. An amplicon of 2539 bps indicated a successful knockout generating the ΔCg_1014 strain compared to the 3880 bps amplicon in ATCC 13032 with intact GT-39. The final, positive clone was further confirmed for the loss of POM by ConA lectin blotting (Fig. 1), with any residual ConA reactivity in the POM deficient strain being attributed to mannosylated lipids and/or lipoarabinomannan (LAM) enriched in the membrane fractions via their resistance to proteinase K digestion (Fig. S3).

**Fig. 2:**
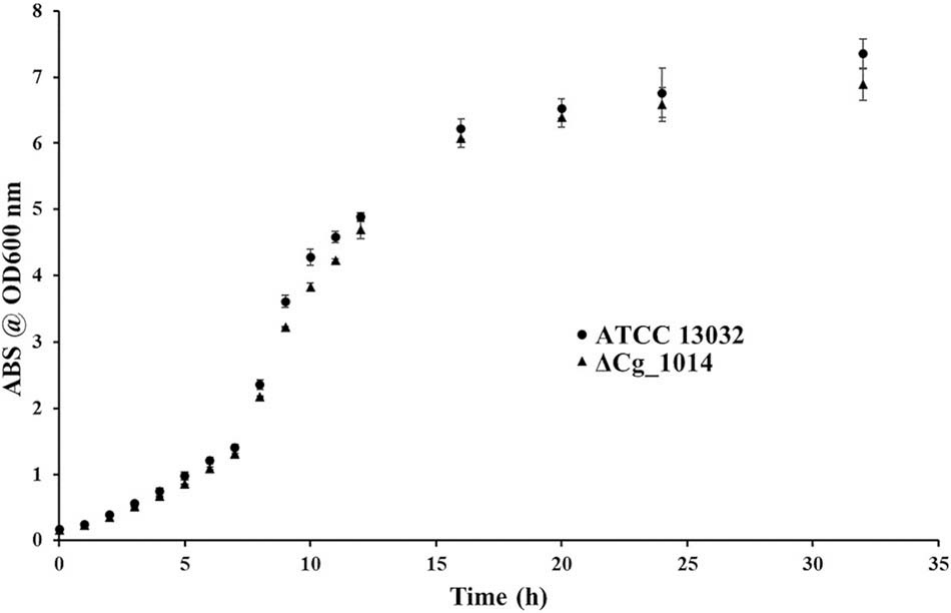
**Effects of GT-39 inactivation on the growth of *C. Glutamicum* ΔCg_1014.** No significant differences to the ATCC 13032 strain were evident at any stage of growth in the ΔCg_1014 strain.

**Fig. 3:**
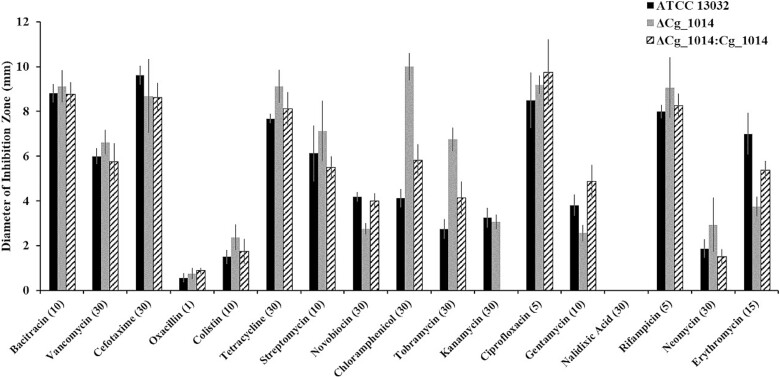
**Sensitivity of *C. Glutamicum* ΔCg_1014 and ΔCg_1014:Cg_1014 to a range of antibiotics by disc diffusion.** Changes to antibiotic sensitivity in the ΔCg_1014 strain were assessed by diameter (in mm) of inhibition zone. The antibiotics having the largest effect on the mutant on ZOI (mm) were tetracycline (30 μg), chloramphenicol (30 μg), tobramycin (10 μg), and erythromycin (15 μg). These antibiotics all inhibit intracellular targets. Complementation of the mutant by Cg_1014 restored sensitivity or resistance to most antibiotics except for chloramphenicol, tobramycin, and erythromycin. These antibiotics target various ribosomal subunits (23S, 30S/50S, and 50S, respectively). Kanamycin resistance of the complemented mutant is conferred by the expression plasmid for antibiotic selection.

The interruption of POM in other actinobacteria has been reported to be non-essential and lacking a discernable phenotype (C. F. Liu et al. 2013; Mahne et al. 2006). This was confirmed with the GT-39 deficient strain of *C. glutamicum* as there were no significant differences in laboratory growth between ATCC 13032 and the ΔCg_1014 mutant (Fig. 2). As previous proteomic studies of actinobacteria have identified that many mannoproteins are either membrane-bound or membrane-associated (He and De Buck 2010; Saxena et al. 2023), the ΔCg_1014 mutant was further screened for a distinct phenotype with antibiotics targeting either membrane-bound/associated or intracellular components – therefore requiring active or passive transport through the membrane. While no obvious patterns were evident, some minor differences in antibiotic susceptibility between the two strains were observed (Fig. 3). Complementation of the ΔCg_1014 strain with pCGE-31 harbouring the native Cg_1014 gene resulted in antibiotic susceptibilities much like the ATCC 13032 strain. Compared to the ATCC 13032 strain, the complemented mutant was still more susceptible to chloramphenicol (30 μg), tobramycin (10 μg).

### In vivo O-Mannosylation

The traditional biochemical characterization of multipass transmembrane proteins is notoriously difficult and problematic (Lehrer et al. 2007; Kaur and Bachhawat 2009). Detection and recovery of actinobacterial GT-39 s produced recombinantly in both the ATCC 13032 and ΔCg_1014 strain was not possible. For this reason, functionality of the recombinantly expressed actinobacterial GT-39 s was assessed via any changes in the *O*-mannosylation of the native *C. glutamicum* mannoproteome both in the WT and POM deficient strains. Limited recapitulation of POM was observed when the mutant strain was complemented with the *C. glutamicum* GT-39 (ΔCg_1014:Cg_1014), with no visible complementation by either the *C. fimi* GT-39 (ΔCg_1014:Celf_3080), or the *C. flavigena* PMT (ΔCg_1014:Cfla_0843) being detected (Fig. 1). The lack of complementation in the mutant strain complemented with site-direct mutants of the *C. glutamicum* GT-39 – mutating conserved residues D^65^N and E^66^Q – suggests that these residues are necessary for enzymatic activity, but not necessarily catalysis (Fig. 1). Like the *S. cerevisiae* and *S. coelicolor* GT-39 s, it is likely that these residues assist in the coordination of an active site divalent Mn2^+^ cation – based on their structural similarity to the GT-66 oligosaccharyltransferase from *Campylobacter lari*, PglB, where activity is metal dependent (Lizak et al. 2011; Bai et al. 2019; Holman et al. 2021).

**Fig. 4:**
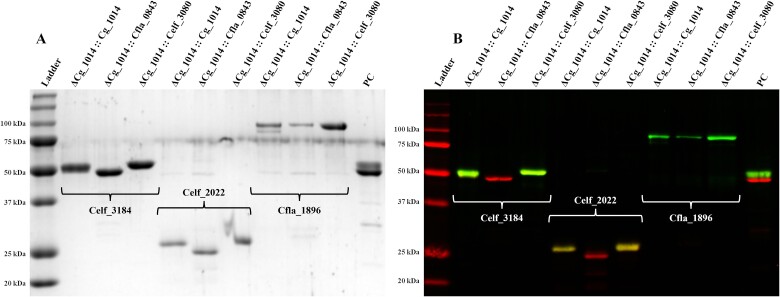
**Coomassie stained 15% SDS-PAGE (A) and ConA-FITC and anti-HIS-Alexa647 blot of recombinant target mannoproteins recovered from spent media of *C. Glutamicum* ΔCg_1014 expressing actinobacterial GT-39 s.** Coomassie stained gel showing target mannoproteins purified from spent media of ΔCg_1014 *C. Glutamicum* expressing *O*-mannosylation operons (A). As direct detection of recombinant GT-39 s was not possible, Celf_3184, Celf_2022, and Cfla_1896 were used as in vivo readouts of POM activity. When co-expressed with each actinobacterial GT-39 in the ΔCg_1014 strain both the *C. Glutamicum* and *C. Fimi* GT-39 s accepted all target mannoproteins as a substrate, with the *C. Fimi* GT-39 producing a more homogenously mannosylated product. The GT-39 of *C. Flavigena* did not glycosylate either of the *C. Fimi* targets, but readily accepted its own cognate target of *O*-mannosylation Cfla_1896. Molecular weight standards are the bio-rad all blue ladder.

**Fig. 5:**
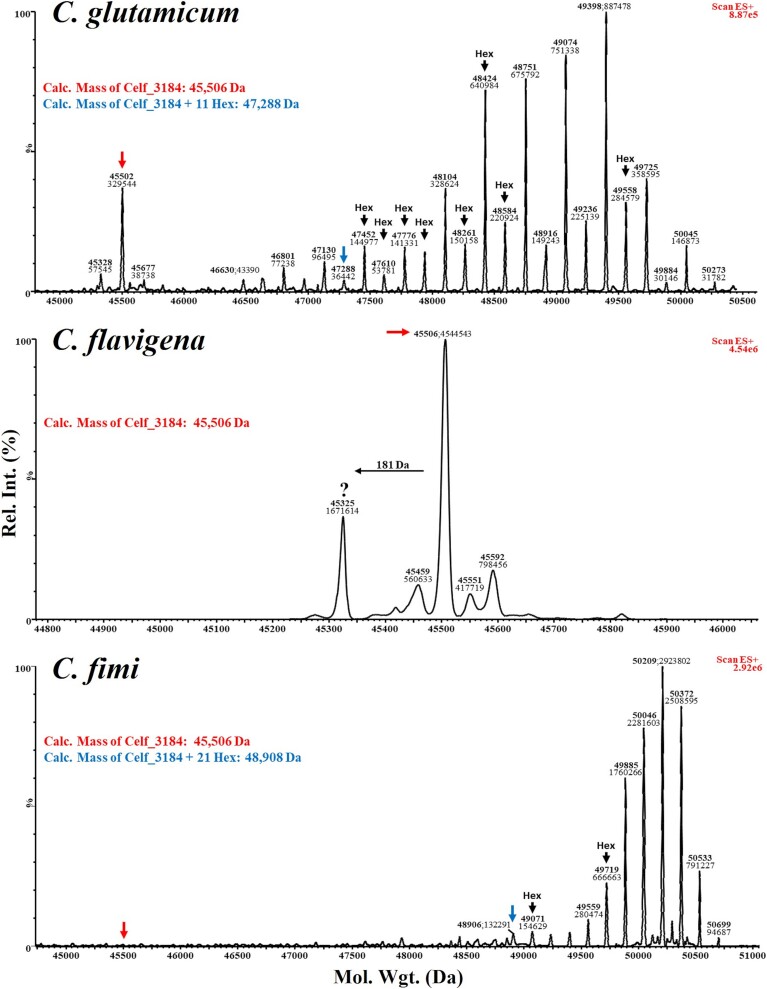
**Hexose modifications by intact mass LC–MS analysis of Celf_3184 expressed in the ΔCg_1014 strain of *C. Glutamicum* using *O*-mannosylation operons complemented with GT-39 s from *C. Glutamicum* (Cg_1014, top panel), *C. Flavigena* (Cfla_0843, middle panel), and *C. Fimi* (Celf_3080, bottom panel).** All recombinant target proteins were recovered from the spent culture media. The calculated mass (with signal peptide removed and no hexose modifications) of Celf_3184 is 45,506 Da which matches with our previous analyses. When expressed alongside Cg_1014, the observed mass profile suggests this protein is modified with between 8–28 hexoses which is also consistent with our previous analyses. With Celf_3080 (the cognate GT-39) a more uniform distribution of hexose modifications was observed, 21–32. Cfla_0843 does not accept this substrate as a target of POM as no mass increase corresponding to a hexose addition was evident.

**Fig. 6:**
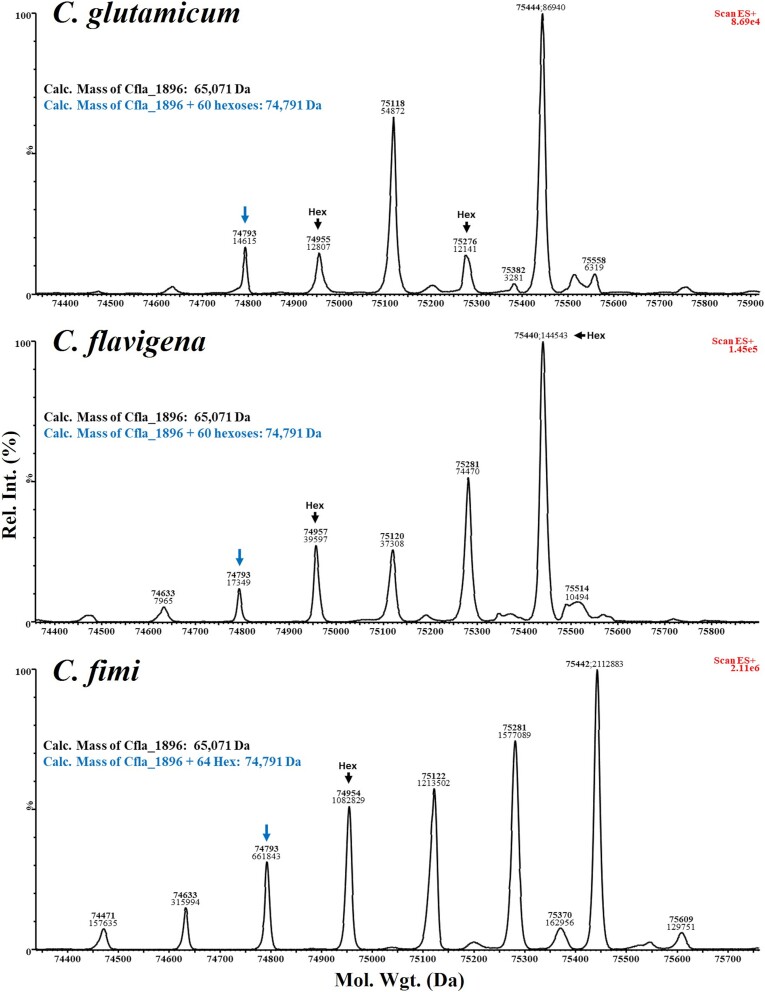
**Hexose modifications by intact mass LC–MS analysis of Cfla_1896 expressed in the ΔCg_1014 strain of *C. Glutamicum* using *O*-mannosylation operons complemented with GT-39 s from *C. Glutamicum* (Cg_1014, top panel), *C. Flavigena* (Cfla_0843, middle panel), and *C. Fimi* (Celf_3080, bottom panel).** All recombinant target proteins were recovered from the spent culture media. The calculated mass (with signal peptide removed and no hexose modifications) of Cfla_1896 is 65,071 Da. This target mannoprotein was equivalently modified with hexoses by all three actinobacterial GT-39 s.

**Fig. 7:**
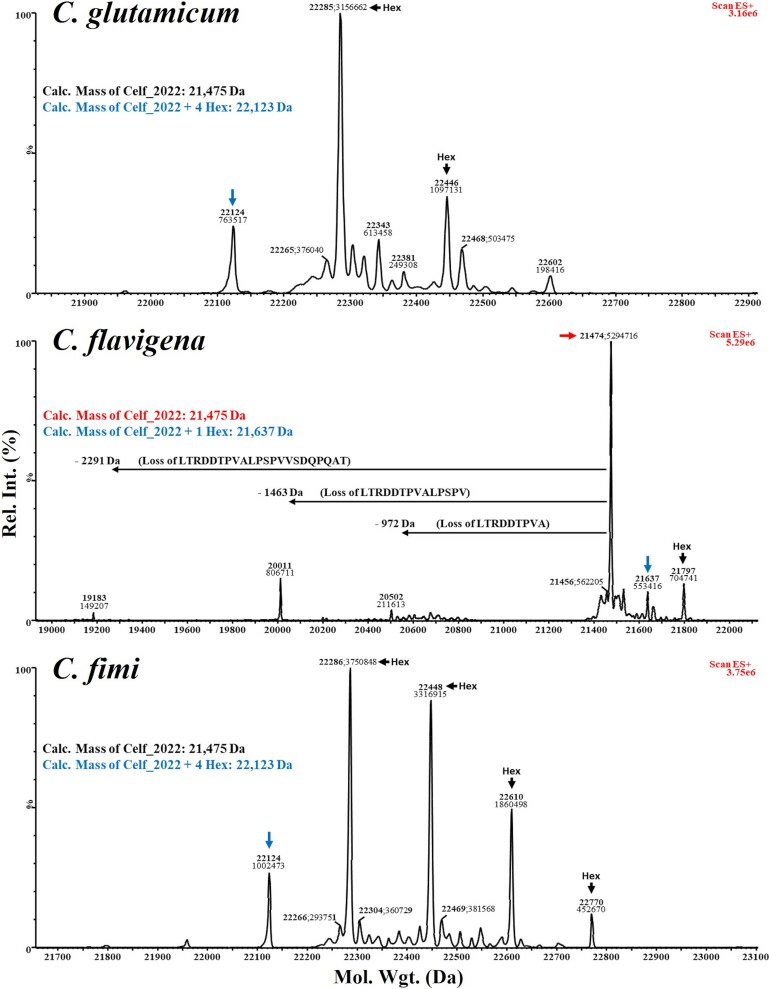
**Hexose modifications by intact mass LC–MS analysis of Celf_2022 expressed in the ΔCg_1014 strain of *C. Glutamicum* using *O*-mannosylation operons complemented with GT-39 s from *C. Glutamicum* (Cg_1014, top panel), *C. Flavigena* (Cfla_0843, middle panel), and *C. Fimi* (Celf_3080, bottom panel).** All recombinant target proteins were recovered from the spent culture media. The calculated mass (with signal peptide removed and no hexose modifications) of Celf_2022 is 21,475 Da which matches with our previous analyses. When expressed alongside Cg_1014, the observed mass profile suggests this protein is modified with between 4–7 hexoses which is also consistent with our previous analyses. With Celf_3080 (the cognate GT-39) a greater degree of hexose modifications was observed, 3–8. Cfla_0843 minimally modifies this target protein with up to 2 hexoses, with a number of suspected *N*-terminal degradation products evident.

As the native *C. glutamicum* mannoproteome proved to be a poor target for assessing the functionality of the *C. fimi* and *C. flavigena* GT-39 s, identified mannoproteins (Celf_3184, Celf_2022, and Cfla_1896) from these species were expressed in the ΔCg_1014 strain alongside each actinobacterial GT-39 in a synthetic operon to better investigate their in vivo activities. Both Celf_3184 (a secreted and highly glycosylated endoglucanase belonging to the Carbohydrate Active Enzymes (CAZy) database family of Glycoside Hydrolase (GH) 6 (Drula et al. 2022), previously known as CenA) and Celf_2022 (a putative cyclophilin type peptidyl-prolyl cis-trans isomerase) have been previously shown to be accurately secreted and mannosylated in the ATCC 13032 strain (Saxena et al. 2023), while Cfla_1896 (also a secreted GH6 member, orthologous to Celf_3184) was identified to be glycosylated during the secretomic analysis of *C. flavigena* (unpublished data). Both GH6s are comprised of a GH6 catalytic module and a CBM2a module, with an intervening linker where the glycosylation takes place. Interestingly, differential mannosylation of these secreted target mannoproteins was detected by ConA lectin blotting when produced in the *O*-mannosylation operons (Fig. 4) and intact mass analysis by LC–MS results confirmed these findings (Figs 5–7). The number of hexose modifications identified on each target mannoprotein by the actinobacterial GT-39 s are summarized in Table 1. The GT-39 s of *C. glutamicum* and *C. fimi* accepted and accurately mannosylated all the target proteins, with subtle differences in the range of hexose modifications. However, the *C. flavigena* PMT demonstrated a distinct preference for its own cognate target of POM, not accepting either of the target mannoproteins from *C. fimi*.

**Table 1:** Summary of hexose modifications by recombinant actinobacterial GT-39 s on secreted target mannoproteins produced in *C. Glutamicum* ATCC 13032 as determined by LC–MS.

**Target Mannoprotein**	**GT-39**	**Hex**
**Celf_3184**	*C. glutamicum*	8–28
	*C. flavigena*	–
	*C. fimi*	21–32
**Celf_2022**	*C. glutamicum*	4–7
	*C. flavigena*	0–2
	*C. fimi*	3–8
**Cfla_1896**	*C. glutamicum*	58–64
	*C. flavigena*	58–64
	*C. fimi*	58–65

### Leader sequence predictions

During this work, the software used to predict the translocon utilized by a particular prokaryotic leader sequence (SignalP sever) was updated. The older iteration, SignalP 5.0, differentiated between Gram-positive, Gram-negative, and archaeal leader sequences and correctly identified the characteristic TAT leader sequence of Celf_3184 with 72.66% likelihood. However, the newer iteration, SignalP 6.0, groups all prokaryotic leader sequences together and predicts the leader sequence of Celf_3184 to be SEC with 99.01% likelihood. For this reason, SignalP 5.0 predictions were used for the entirety of this work.

### Investigating the Celf_2022 leader sequence

Originally, predictive tools were unable to identify an *N*-terminal secretion signal associated with Celf_2022 (a predicted cyclophilin type peptidyl-prolyl cis-trans isomerase) even though it was first identified as a mannoprotein in the spent media of *C. fimi* cultures during a secretomic analysis (Wakarchuk et al. 2016). More recent iterations of the SignalP algorithm now predict the signal peptide of this protein as utilizing a pathway other than SEC or TAT (likelihood of 0.998 using SignalP 5.0), leading to the Celf_2022 secretion signal being denoted as “Other.” To confirm this prediction, the signal peptide of Celf_2022 was replaced by the predicted SEC and TAT signal peptides of Celf_1230 and Celf_3184 respectively. In actinobacteria, these two secretion systems are both known to in be involved in the export of *O*-mannosylated proteins but differ in the conformation of their substrates during translocation; SEC substrates are secreted in an unfolded state while TAT substrates are secreted fully folded. If Celf_2022 was truly a SEC or TAT secreted protein, replacement of its native secretion signal by one from a different protein utilizing the same translocon should not alter the localization or *O*-mannosylation profile of the mature protein. Conversely, using a non-native translocon may not necessarily alter the localization of Celf_2022, but could alter or completely abolish the *O*-mannosylation profile due to the way it is presented to the GT-39 (folded vs. unfolded).

**Fig. 8:**
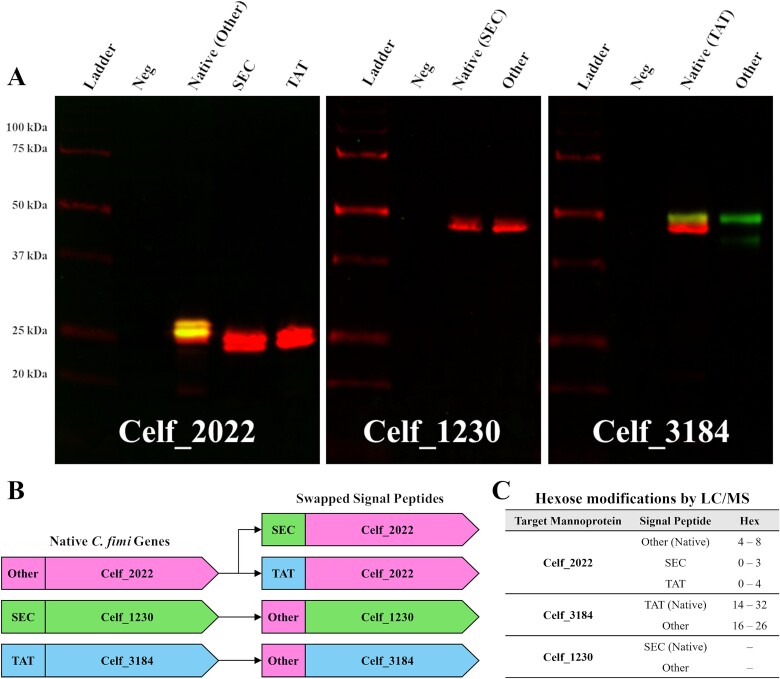
**ConA-FITC and anti-HIS-Alexa647 blots of swapped signal peptide constructs enriched from spent cultures of *C. Glutamicum* ATCC 13032 (A). Schematic of swapped leader constructs (B). Summary of hexose modifications per construct as identified by LC–MS (C).** When lead by its native “other” leader sequence, Celf_2022 is both secreted and mannosylated in *C. Glutamicum* ATCC 13032. Replacement of this “other” leader sequence by a SEC or TAT signal peptide retains secretion but dramatically diminishes mannosylation. Replacement of the SEC signal peptide of Celf_1230 by the “other” leader sequence results in no change to secretion or mannosylation pattern. Replacement of the TAT signal peptide of Celf_3184 by the “other” leader sequence results in a more homogenous glycoform (A). Molecular weight standards are the bio-rad all blue ladder. Schematic representation of swapped signal peptide constructs, using secreted proteins from *C. Fimi* and their respective signal peptides (B). LC–MS analysis confirmed that both SEC and TAT translocation of Celf_2022 reduces the total number of hexose modifications compared to the unknown secretion system, while Celf_3184 is more uniformly *O*-mannosylated and the glycosylation status of Celf_1230 does not change using the unknown secretion system (C).

When produced in *C. glutamicum* ATCC 13032 with its native signal peptide, Celf_2022 is mannosylated and exported as previously reported. However, replacement of this unclassified signal peptide by either the classical SEC (from Celf_1230) or TAT (from Celf_3184) signal peptide resulted in *secreted* Celf_2022 that was primarily non-glycosylated. The minimal amount of glycosylated Celf_2022 secreted using either SEC or TAT signal peptides showed approximately half the number of total hexose modifications compared to when Celf_2022 was secreted using its native signal sequence (Fig. 8, S4). Replacement of the native SEC and TAT leaders of Celf_1230 and Celf_3184, respectively, by the unknown leader of Celf_2022 did not change the localization or mannosylation status of either protein, however, a narrower distribution of hexose modifications was observed on Celf_3184 when secreted via using the signal peptide of Celf_2022 (Fig. 8, S5).

## Discussion

### *C. Glutamicum* GT-39 is dispensable under laboratory conditions

Inactivation and complementation of the gene encoding the sole GT-39 of *C. glutamicum* has been reported previously, however only the presence or absence of mannosylation on secreted proteins was assayed; the more abundant membrane associated or cytoplasmic mannoproteins were not considered (Mahne et al. 2006). In addition, no phenotype or growth characteristics of this knockout have been reported to date. For this purpose, the inactivated truncation mutant which maintains up- and downstream effectors (Fig. S1B) for Cg_1013 – a hypothetical protein with unknown function – and Cg_1015 – a uroporphyrin-III C/tetrapyrrole (corrin/porphyrin) methyltransferase (Kalinowski et al. 2003) – was designed to be a more refined approach towards generating the mutant required for the investigation of POM in *C. glutamicum*.

Inactivation of the *C. glutamicum* GT-39 results in no discernable growth phenotype (Fig. 2) exemplifying the seemingly dispensable nature of this modification in actinobacteria under laboratory conditions, much like what was observed in the *M. tuberculosis* GT39 knockout (C. F. Liu et al. 2013). In addition, there was no observable morphological differences between the strains, although some minor differences in antibiotic susceptibility between ATCC 13032 and ΔCg_1014 were seen with some antibiotics primarily targeting Gram positives and all with intracellular targets. These antibiotics all require transport (passive or active) across the cell membrane, so these results suggest that POM may affect either cell wall permeability or specific uptake mechanisms. In *S. coelicolor*, non-functional PMT mutants displayed only mild increases to antibiotic susceptibility against vancomycin and β-lactams (Howlett et al. 2018). These increased susceptibilities were not observed with the ΔCg_1014 strain, suggesting that in *C. glutamicum*, major cell wall effects are not a phenomenon associated with a lack of protein mannosylation. Complementation of ΔCg_1014 resulted in incomplete restoration of these antibiotic sensitives. The cause of the changes in antibiotic sensitivity might be cell permeability, or target modification. Currently we have no explanation for this, but there is some precedence for the antibiotic targets, ribosomal subunits, in *M. tuberculosis* carrying hexose modifications (Birhanu et al. 2019).

### Differential complementation of ΔCg_1014 by Actinobacterial GT-39 s

We were unable to detect recombinant GT-39 in any construct even though all strains were confirmed to harbour the correct vector and insert by plasmid rescue into *E. coli*. Integral membrane proteins can be notoriously difficult to detect, enrich, and biochemically characterize (Lehrer et al. 2007; Kaur and Bachhawat 2009) as they are often present at very low levels and lose their native activity and the conformation required for enzymatic activity when removed from biological membranes (Kongpracha et al. 2022; Liu et al. 2022; Tiefenauer and Demarche 2012). In *S. coelicolor*, evidence has been presented that even very conservative single amino acid substitutions to the native GT-39 sequence may cause misfolding and rapid degradation leading to both a lack of activity and detection by Western blotting (Holman et al. 2021).

Complementation with the *C. glutamicum* GT-39 lead to restoration of the expression of the mannoproteome, but it did not look exactly like the wildtype sample. Simple complementation with heterologous GT-39 s also failed to restore the native mannoproteome. This suggested to us that these closely related GT-39 s may display specificity for their native targets of POM, the possibility of these strains producing no active *Cellulomonas* GT-39 could not be ruled out. For this reason, identified mannoproteins from *C. fimi* and *C. flavigena* were used as recombinant reporter proteins for the in vivo readout of POM by these actinobacterial GT-39 s expressed in the ΔCg_1014 strain of *C. glutamicum*.

We also investigated the conserved “DE motif” seen in all GT-39 proteins and observed loss of complementation as was seen in *S. coelicolor* (Holman et al. 2021). Mutation of either D^65^N or E^66^Q totally abolished the activity of the *C. glutamicum* GT-39 (Fig. 1). Although, as mentioned above this may also be due to lack of protein expression due to instability from these mutations.

### In vivo Mannosylation of heterologous target proteins

#### Celf_3184

The Celf_3184 protein is heavily mannosylated in both its native organism and the recombinant host *C. glutamicum* (Saxena et al. 2023), so we chose this target mannoprotein to assess and compare the in vivo activities of the different actinobacterial GT-39 s expressed in *C. glutamicum*. Importantly, POM activity was detected in the mutant strain producing the *C. fimi* GT-39, meaning that it too is produced in an active form and localized correctly. Logically, the *C. fimi* GT-39 readily accepts its cognate substrate and mannosylates it much more uniformly than what was observed with the *C. glutamicum* GT-39 (see Table 1, Fig. 5). As this initial attachment of mannose to S/T residues is the limiting step in POM these results show that the *C. fimi* GT-39 preferentially recognizes and acts on a greater number of the *O*-mannosylation sites contained in Celf_3184 than did the *C. glutamicum* GT-39. However, the *C. flavigena* GT-39 does not appear to accept this protein as a target for POM (Figs 4 and 5, Table 1).

#### Cfla_1896

*C. flavigena* harbours its own orthologues of the *C. fimi* Celf_3184. Interestingly, these three orthologues (UniProt: D5UEY3, D5UKD4, D5UKD5) all contain a longer linker region corresponding to the identified glycopeptide of Celf_3184 (UniProt: F4GZY2) suggesting they may be natively *O*-mannosylated. To validate the in vivo activity of the recombinantly produced *C. flavigena* GT-39 and to test the hypothesis that a structural difference in these orthologous GH-6 targets resulted in the lack of POM activity observed, Cfla_1896 was incorporated into the *O*-mannosylation operon. Cfla_1896 is also the most unique GH-6 of *C. flavigena*, containing the longest linker (69 aa), an inverted domain architecture compared to Celf_3184 (C-terminal CBM2 for the former and N-terminal CBM2 for the latter), and a greater number of S/T residues in the linker to be potentially *O*-mannosylated.

When expressed in the POM deficient strain of *C. glutamicum* with its cognate GT-39, Cfla_1896 was both secreted and heavily mannosylated with up to 65 hexose units, confirming that active *C. flavigena* GT-39 was produced, correctly inserted into the membrane, and able to recognize its cognate protein – further validating the platform for the study of actinobacterial POM. It is odd that Cfla_1896 was mannosylated to a similar degree by all three actinobacterial GT-39 s indicating that the GT-39 s of *C. fimi* and *C. glutamicum* may have more relaxed substrate preferences while the *C. flavigena* enzyme is stricter in its acceptance of target proteins and may display a strong preference for native substrates. The longer linker region of Cfla_1896 could make its acceptor site good for any GT-39. This suggests such a protein will be of value in further structure–function studies of the various GT-39 enzymes.

#### Celf_2022

Celf_2022 was included in the *O*-mannosylation operon as a suitable reporter target protein with a different protein fold for recombinant expression, secretion, and POM in *C. glutamicum* ATCC 13032 (Saxena et al. 2023). Much like Celf_3184, the *C. glutamicum* and *C. fimi* GT-39 s readily accepted Celf_2022 as a target of POM and in both cases the total number of hexoses added agreed with what was observed when the mannoprotein was identified in the secretome of *C. fimi* (Saxena et al. 2023). When produced in the *O*-mannosylation operon in concert with the *C. flavigena* GT-39, most of the secreted protein was non-glycosylated and the small amount of material that was *O*-mannosylated had a maximum of two hexose modifications. The glycopeptide of Celf_2022 comprises the N-terminal 24 aa of the mature polypeptide (following the removal of the 53 aa other-type signal sequence) and is lacking in defined structure (UniProt: F4H0A5). While it is currently uncertain if this protein is secreted in a folded or unfolded state, the length of the unstructured, mature N-terminus appears to be a poor substrate for the *C. flavigena* GT-39 as evidenced by the minimal amount of POM observed when these two proteins are expressed together in the operon.

### Secretion Translocons also influence POM

There had been a proposal 20 years ago that bacterial POM required secretion through the SEC translocon (VanderVen et al. 2005). Our observations show that is likely not the case. Replacing the signal peptide of Celf_2022 with that of either a traditional SEC or TAT secreted substrate dramatically alters the POM profile of the mature protein but not its localization when expressed in *C. glutamicum* ATCC 13032 (Fig. 8A). The native Celf_2022 could be secreted by a translocon independent of both SEC and TAT as only its native signal peptide results in mature Celf_2022 with a POM profile matching that of the original protein identified from *C. fimi* (Saxena et al. 2023).

Celf_1230 is not a mannosylated protein (Wakarchuk et al. 2016) and its lack of mannosylation profile does not change by ConA lectin blot when the native SEC leader is replaced by the leader sequence of Celf_2022 (Fig. 8A). However, when the native TAT leader of Celf_3184 is replaced by the Celf_2022 leader sequence a narrower mannosylation pattern was observed on the secreted and enriched GH-6 resulting in 16–26 hexoses as opposed to 14–32 hexoses (Saxena et al. 2023) and no non-mannosylated material was observed by either His tag immunoblotting or LC–MS (Figs 8A and C, S5). These hexose modifications can be solely attributed to the *C. glutamicum* native GT-39, as expressing these constructs in the ΔCg_1014 mutant produced non-mannosylated protein. This narrower range of hexose modifications may therefore show a unique association between the unknown secretion system and GT-39 enzyme. The secretion system utilized by Celf_2022 does not result in the aberrant mannosylation of natively non-mannosylated proteins and can produce accurately modified non-native mannoproteins.

Currently, the translocon utilized by Celf_2022 is unknown. At present it cannot be clearly discerned if this is a novel actinobacterial secretion system or one that is already known. Importantly, this experiment strengthens the argument that there is more than just SEC-dependant POM occurring in actinobacteria. To truly dissect how this secretion system interacts with and influences POM, it must first be classified. Of note among potential candidates is the recently identified class of non-classically secreted proteins lacking traditionally identifiable *N*-terminal signal peptides. Currently, it cannot be entirely ruled out that Celf_2022 may be a member of this class of proteins. While the study of non-classical secretion in bacteria is steadily growing – with reports in *Bacillus*, *Listeria*, *Staphylococcus*, *Streptococcus*, *Mycobacterium*, and others (Naclerio et al. 1995; Lei et al. 2000; Schaumburg et al. 2004; Målen et al. 2007; He and De Buck 2010; Pasztor et al. 2010) – the relationship between this novel secretion system and protein glycosylation in bacteria is not known.

## Conclusion

While POM has been known to occur in various actinobacterial species, a significant lack of information still exists about how this modification benefits them outside of the context of virulence. Even though they are closely related, this work demonstrated a tangible difference in the functionality of actinobacterial GT-39 s. We have demonstrated that we have a specific means to recombinantly express a GT-39 and a cognate reporter protein, which in the absence of classical biochemical assays allows a more detailed study of structure/function relationships. Moving forward, we expect to be able to ask questions about preferred or engineered glycosylation sites and further mutational analysis of the GT-39 itself.

Of note is the additional evidence that POM is not solely SEC dependent in actinobacteria; POM can also occur via the TAT pathway and the currently unidentified secretion pathway utilized by Celf_2022. These findings showcase how poorly understood actinobacterial POM truly is, both in how it is performed and what impact it has on the organisms that are capable of it.

Of course there are some limitations in our study, like the lack of detection of the expression levels of the recombinant GT-39 enzymes. The lack of complete complementation suggests the expression levels are not like the native organism, and we will have to find a way to quantify the expression going forward. We know that these enzymes are made through the reporter proteins but differences in expression could not quantitated, which could make further mutational studies difficult. Another limitation is that the secretion pathway(s) appears to be involved in target protein selectivity based on the protein being exported. Future work will involve investigations to find suitable native mannoproteins are so that we can determine if this is selectivity is limited to heterologous protein expression. In addition, manipulation of the secretion pathways might help define the “other” pathway we are proposing for Celf_2022.

## Methods

### Media, strains, and expression conditions

All strains were grown in 2YT media (16 g/L tryptone, 10 g/L yeast extract, 5 g/L NaCl, BioShop Canada). NEB® Stable *E. coli* (NEB) was used for routine cloning and plasmid production. BL21 (DE3) *E. coli* and *C. glutamicum* ATCC13032 were used for recombinant protein production. Electrocompetent *C. glutamicum* were cultured in MBGT media (16 g/L tryptone, 10 g/L yeast extract, 5 g/L NaCl, 35 g/L glycine, 0.1% Tween-80) and outgrowths were performed in 2YT + 91 g/L sorbitol.

Single colonies of *C. glutamicum* expression constructs were inoculated into 25 mL 2YT containing 50 μg/mL kanamycin and 25 μg/mL nalidixic acid, then incubated overnight at 30 °C with shaking at 180 RPM. The following day, overnight cultures were diluted into 250 mL 2YT expression cultures (containing 50 μg/mL kanamycin and 25 μg/mL nalidixic acid) to an OD_600_ ≈ 0.1 and incubated at 30 °C, 180 RPM. Cultures were induced with 0.5 mM IPTG once they reached an OD_600_ ≈ 0.6 and allowed to express overnight at 30 °C. Following induction, cultures were harvested by centrifugation at 5,000 x *g* for 10 mins at 4 °C.

### *C. Glutamicum* GT-39 knockout generation and characterization

The gene encoding the sole GT-39 in *C. glutamicum* ATCC 13032 (Cg_1014) was replaced with a truncated and inactive mutant via homologous recombination assisted by the pK18mobsacB suicide vector. A synthetic gene composed of the truncated Cg_1014 gene containing only the cytoplasmic N-terminal domain (1–35 aa), the first transmembrane region (36–58 aa), and the extracellular C-terminal domain (506–520 aa) flanked by upstream and downstream regions of 1001 bps was synthesized (IDT), restriction cloned into pK18mobsacB using XbaI and SalI, and subsequently cloned into electrocompetent NEB® Stable *E. coli* (NEB). The sequenced knockout construct was transformed directly into electrocompetent *C. glutamicum* ATCC 13032 and selection for gene replacement was carried out using the previously established *sac*B methodology (Schäfer et al. 1994) with counterselection in the presence of 20% sucrose. Replacement of Cg_1014 with the truncated gene was confirmed via PCR with specific flanking primers (Table S2).

Growth curves were performed in triplicate in 2YT media (containing 50 μg/mL kanamycin and 25 μg/mL nalidixic acid) at 30 °C and 180 RPM throughout and OD_600_ was measured spectrophotometrically. Antibiotic susceptibility was assayed with the Kirby-Bauer (KB) methodology at 30 °C using the following antibiotic discs in quadruplicate: bacitracin (BAC) 10 U, vancomycin (VAN) 30 μg, cefotaxime (CTX) 30 μg, oxacillin (OXA) 1 μg, colistin (COL) 10 μg, tetracycline (TET) 30 μg, streptomycin (STR) 10 μg, novobiocin (NOV) 30 μg, chloramphenicol (CHL) 30 μg, tobramycin (TOB) 10 μg, kanamycin (KAN) 30 μg, ciprofloxacin (CIP) 5 μg, gentamicin (GEN) 10 μg, nalidixic acid (NAL) 30 μg, rifampicin (RIF) 5 μg, neomycin (NEO) 30 μg, and erythromycin (ERY) 15 μg (Fisher).

### Vector and Mannosylation operon design and construction

The *E. coli/C. glutamicum* shuttle vector pTGR-5 (Ravasi et al. 2012) was received as a generous gift from Dr. Pablo Ravasi. To generate the high-level expression plasmid, pCGE-31, the Ptac region in pTGR-5 (XbaI – NheI fragment) was replaced with the lac UV5 + tandem Plac system from the expression vector pCW (Janesch et al. 2019) utilizing synthetic primers (Table S2) and maintaining the *sod* RBS and spacing already present in pTGR-5.

GT-39 genes from *C. glutamicum* ATCC 13032, *C. fimi* ATCC 484, and *C. flavigena* ATCC 482 were amplified from genomic DNA using specific primers containing NdeI (5′) and HindIII (3′) restriction sites (Table S2). The triple lac operator from pCW-MalET (Janesch et al. 2019) was used to replace the single lac operator in pTGR-5 (Ravasi et al. 2012) using synthetic primers containing BamHI (5′) and NheI (3′) restriction sites (Table S2) while maintaining the RBS_sod_ and nucleotide spacing, generating the pCGE-31 shuttle vector (Fig. S6A) for the recombinant expression of actinobacterial GT-39 s in *C. glutamicum*. A synthetic operon used to assay POM in vivo using a known mannoprotein (Fig. S6B) was designed and inserted upstream of the rrnB T1 terminator using NdeI (5′) and AvrII (3′) containing actinobacterial GT-39 s followed by a single lac operator, RBS_sod_, and actinobacterial mannoprotein Celf_3184. Constructs were confirmed via restriction digest and sequencing, transformation of constructs into *C. glutamicum* was confirmed via plasmid rescue into NEB® Stable *E. coli* (NEB).

### Electrocompetent *C. Glutamicum*

A single colony of *C. glutamicum* ATCC13032 or ΔCg_1014 was inoculated into 50 mL 2YT containing 25 μg/mL nalidixic acid and incubated overnight at 30 °C with shaking at 180 RPM. The following day, 1 L MBGT containing 25 μg/mL nalidixic acid was inoculated to an OD_600_ ≈ 0.1 using the overnight culture and incubated at 30 °C, 180 RPM. When the OD_600_ ≈ 0.25–0.25 (about 2 h) 0.5 μg/mL ampicillin was added and the culture was allowed to continue incubating at 30 °C, 180 RPM for an additional 1.5 h.

Following incubation, cells were harvested at 5,000 x *g* for 10 mins at 4 °C. Cells were resuspended in 150 mL 10% glycerol and centrifuged at 5,000 x *g* for 10 mins at 4 °C a total of 3 times. Final cell pellets were resuspended in 10% glycerol to a final OD_600_ ≈ 200 and aliquots of 100 μL were stored at −80 °C.

Electrocompetent *C. glutamicum* ATCC 13032 and ΔCg_1014 were transformed with an adapted protocol (Van der Rest et al. 1999; Ruan et al. 2015). Competent cell aliquots were thawed on ice and allowed to incubate with 750 ng DNA for 10 mins prior to transformation. Cells and DNA were electroporated in 0.2 cm cuvettes using a BioRad Gene Pulser Mini at 2.5 kV for 4.80–5.20 ms. Immediately following, 1 mL 2YT + 91 g/L sorbitol was added to cells and outgrowths were placed at 46 °C for 6 mins to inactivate the host restriction system and increase transformation efficiency. Cells were allowed to recover at 30 °C, 180 RPM for 2 hours. Cells were harvested via centrifugation at 5000 x *g* for 1 min and resuspended in 200 μL fresh outgrowth medium prior to plating on agar containing 50 μg/mL kanamycin and 25 μg/mL nalidixic acid. Plates were incubated at 30 °C for 48–72 hours until colonies appeared.

All constructs in *C. glutamicum* were confirmed via plasmid rescue in *E. coli*. Miniprepped plasmid DNA from *C. glutamicum* constructs was transformed into electrocompetent *E. coli* for propagation, then confirmed by restriction digest and sequencing.

### Purification of secreted HIS_6_-tagged proteins

Cell pellets were harvested by centrifugation at 5,000 x *g* for 10 mins at 4 °C and stored at −20 °C. The spent culture media was clarified via centrifugation at 20,000 x *g* for 30 mins at 4 °C and particulates were removed with a 0.45 μm PES bottle top filter (supp). A 10X stock of HisTrap A Buffer (1 M HEPES, 3 M NaCl, pH 8.0) was diluted to 1X with the clarified spent media, bringing the mixture to a final concentration of 100 mM HEPES, 300 mM NaCl, pH 8.0 prior to affinity chromatography.

All recombinantly produced proteins were enriched via affinity chromatography using Roche cOmplete™ His-Tag Purification Resin (Millipore-Sigma) and chromatography on an AKTA Start (Cytiva). Clarified spent media were loaded onto an equilibrated XK-16 column (Cytiva) containing 15 mL cOmplete™ resin with HisTrap A buffer at a flowrate of 2 mL/min. The column was washed with 3 CV of HisTrap A buffer prior to elution along a linear gradient (0–100%) of HisTrap B (100 mM HEPES, 300 mM NaCl, 500 mM imidazole, pH 8.0) over 5 CV. Fractions containing recombinant proteins were pooled, concentrated, and buffer exchanged into 50 mM HEPES, 150 mM NaCl, pH 7.4 using 20 mL VivaSpin concentrators with 10,000 MWCO (Cytiva).

### Isolation of *C. Glutamicum* membrane proteins

Frozen cell pellets were resuspended (1 g/ 10 mL) in ConA buffer A (20 mM Tris, 0.5 M NaCl, 1.0 mM CaCl2 and 1.0 mM MnCl_2_, pH 7.4) with Sigma P-2714 protease inhibitor cocktail. The cells were lysed using an Emulsiflex-C5 (Avestin) at ≥20,000 psi. Following centrifugation at 6,000 x g for 5 min at 4 °C to remove debris, the supernatant was centrifuged again at 20,000 x g for 20 min at 4 °C. The 20,000 x g pellet fraction was resuspended in 0.5 mL of ConA buffer A with 0.1% Triton X-100 and incubated at room temperature, using a tube roller, overnight at 4 °C. Solubilized membranes were centrifuged at 100,000 x g for 1 h at 4 °C and the resultant supernatants were used for Western and lectin blotting.

### Western and lectin blot protocol

Lectin blots were performed as described in the manufacturers data sheets. Briefly, the proteins of interest were separated on a 15% SDS page gel using a miniProtean system (Bio-Rad), which was then rinsed three times for 5 min each in excess Tris buffered saline pH 7.6 (TBS, 50 mM Tris, 150 mM NaCl, pH 7.6) before being blotted to PVDF membrane using a Trans-Blot Turbo transfer system (Bio-Rad). The transfer was performed in 48 mM Tris, 39 mM glycine using 2.5 A, 25 V for 8 mins. The protein-bearing PVDF membrane was then rinsed three times for 5 minutes each, in TBS and blocked for one hour in 5% BSA in TBS at room temp. The blocked membrane was washed three times for 5 minutes in TBS at room temp, then incubated overnight at 4 °C in 0.05% Tween 20; 1 mM CaCl_2_; 1 mM MgCl_2_; 1 mM MnCl_2_; 0.5 μg/mL ConA-FITC conjugated lectin (Millipore-Sigma); and 1:10, 000 AlexaFluor 647 anti-HIS_6_ (Bio-Rad) in TBS. The membrane then underwent three 10-min washes in TBS pH 7.6 at room temp and was visualized on a Bio-Rad ChemiDoc. Molecular weight markers were Bio-Rad All Blue standards.

### Intact mass LC–MS analysis of Celf-3184 Mannosylated by Actinobacterial GT-39 s

Intact mass analysis was performed using an Ultimate 3000 (Dionex/Thermo Fisher Scientific) linked to an LTQ-Orbitrap XL hybrid mass spectrometer (Thermo Fisher Scientific). 5 μg of each protein was injected on to a 2.1 × 30 mm Poros R2 column (Thermo Fisher Scientific) and resolved using the following rapid gradient: hold at 20% mobile phase A for 3 minutes, 20% - 90% mobile phase B in 3 minutes, hold at 90% mobile phase B for 1 minute. Mobile phase A was 0.1% formic acid in ddH2O and mobile phase B was acetonitrile. The flow rate was 3 mL/min with 100 μL split to the electrospray ion source. Optimal peak shape was achieved by heating the column and mobile phase to 80 °C. The mass spectrometer was tuned for small protein analysis using myoglobin and the resolution was set to 15,000. Mass spectra were acquired from m/z 400 to 2000 in the Orbitrap at 1 scan per second. The spectra acquired across the protein peak were summed and deconvoluted using MaxEnt 1 (Waters).

## CRediT taxonomy

Hirak Saxena (Formal analysis [Lead], Investigation [Equal], Methodology [Lead], Visualization [Lead], Writing—original draft [Lead], Writing—review & editing [Lead]), Rucha Patel (Formal analysis [Equal], Investigation [Equal], Validation [Equal]), John Kelly (Formal analysis [Equal], Investigation [Equal], Methodology [Equal], Validation [Equal], Writing—review & editing-Supporting), and Warren Wakarchuk (Conceptualization [Lead], Funding acquisition [Lead], Investigation [Equal], Project administration [Lead], Supervision [Lead], Writing—original draft [Equal], Writing—review & editing [Equal])

## Supplementary Material

Saxena_et_al_supplemental_Rev_6_Glycobiology_2024_cwae095

## Data Availability

Data from the manuscript is available upon request.
