# Supplementary material for: Differential substrate preferences IN ACTINOBACTERIAL protein O-MANNOSYLTRANSFERASES and alteration of protein-O-MANNOSYLATION by choice of secretion pathway
Source: Glycobiology. 2024 Dec 3;35(1):cwae095. doi: 10.1093/glycob/cwae095 (PMC11727336; doi:10.1093/glycob/cwae095)
Supplement: Saxena_et_al_supplemental_Rev_6_Glycobiology_2024_cwae095 [file saxena_et_al_supplemental_rev_6_glycobiology_2024_cwae095.docx]

**DIFFERENTIAL SUBSTRATE PREFERENCES IN ACTINOBACTERIAL PROTEIN *O*-MANNOSYLTRANSFERASES AND ALTERATION OF PROTEIN-*O*-MANNOSYLATION BY CHOICE OF SECRETION PATHWAY**

Hirak Saxena^1^, Rucha Patel^1^, John Kelly^2^, and Warren Wakarchuk^1*^.

1. Department of Biological Sciences, University of Alberta, Edmonton, AB

2. Human Health Therapeutics, National Research Council of Canada, Ottawa, ON

*Corresponding author:

Warren Wakarchuk, email address wakarchu@ualberta.ca

Telephone 1-780-492-0587

Running title: Differential Protein-*O*-Mannosylation by Actinobacterial GT-39s and Secretion Pathways

Key words: actinobacteria, protein-*O*-mannosylation, protein-*O*-mannosyltransferase

Supplementary Data Included: Figs S1 – S6 and Tables S1– S2

**
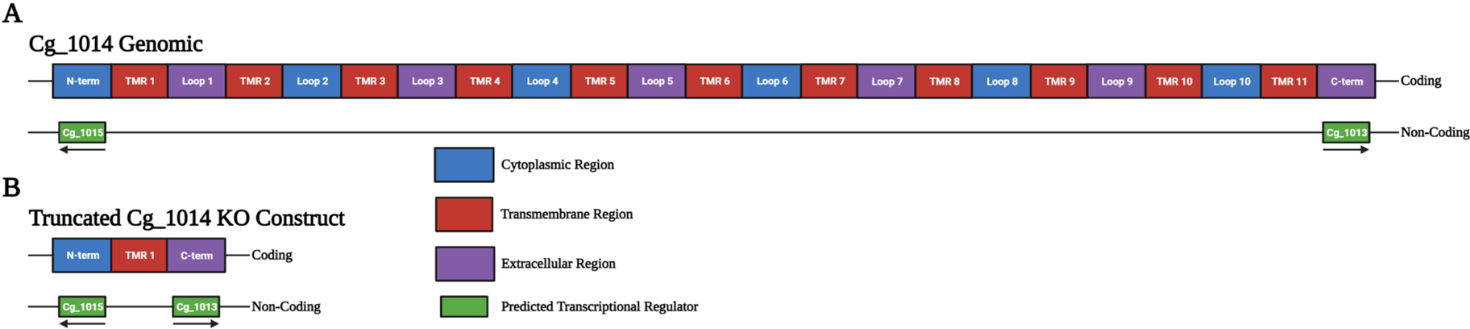
**

**Figure S1: Schematic and predicted topology of genomic Cg_1014 and predicted transcriptional regulators on the non-coding strand (A) maintained in the inactivated knockout mutant (B).** The *C. glutamicum* GT-39 Cg_1014 contains (as predicted by TMHMM-2.0) 11 transmembrane regions (TMR, red), 5 cytoplasmic loops (blue), and 6 extracellular loops (purple). The predicted cytoplasmic N-terminal and extracellular C-terminal domains also contain predicted transcriptional regulators for the neighbouring genes Cg_1013 and Cg_1015 (A). As catalytic activity is thought to be harboured on extracellular Loop 1 a truncated and inactive mutant was designed (B) containing only the N- and C-terminal domains connected by TMR 1, maintaining down- and upstream transcriptional effectors.


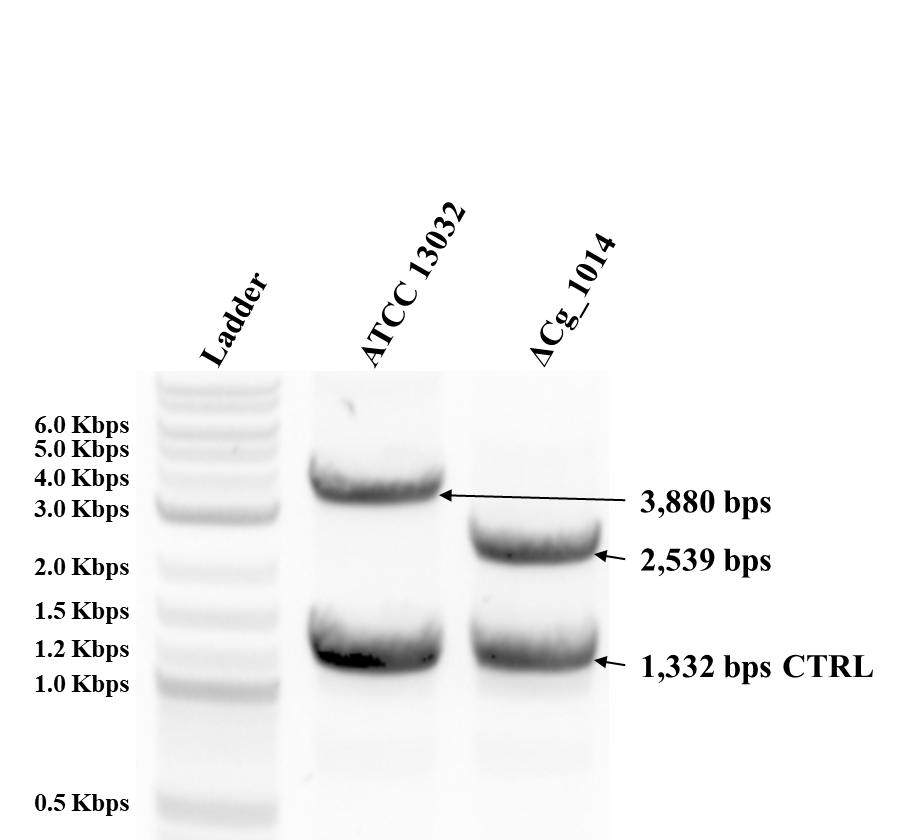


**Figure S2: 0.8% agarose gel showing genomic knockout of GT-39 in *C. glutamicum* ATCC 13032 and ΔCg_1014.** Primers specific to the flanking regions ≈ 1,000 bps up- and downstream of Cg_1014 were used to identify successful homologous recombination events. In ATCC 13032 this amplicon is 3,880 bps and when Cg_1014 is replaced by the truncated inactive sequence the amplicon is 2,539 bps. This decrease exactly corresponds to the 1,341 bps removed from Cg_1014 (Δ59 – 505 aa) to generate the mutant gene. An additional amplicon of 1,332 was also produced using primers specific for the *C. glutamicum* homologue of the maltose binding protein (MBP) as a control.

**
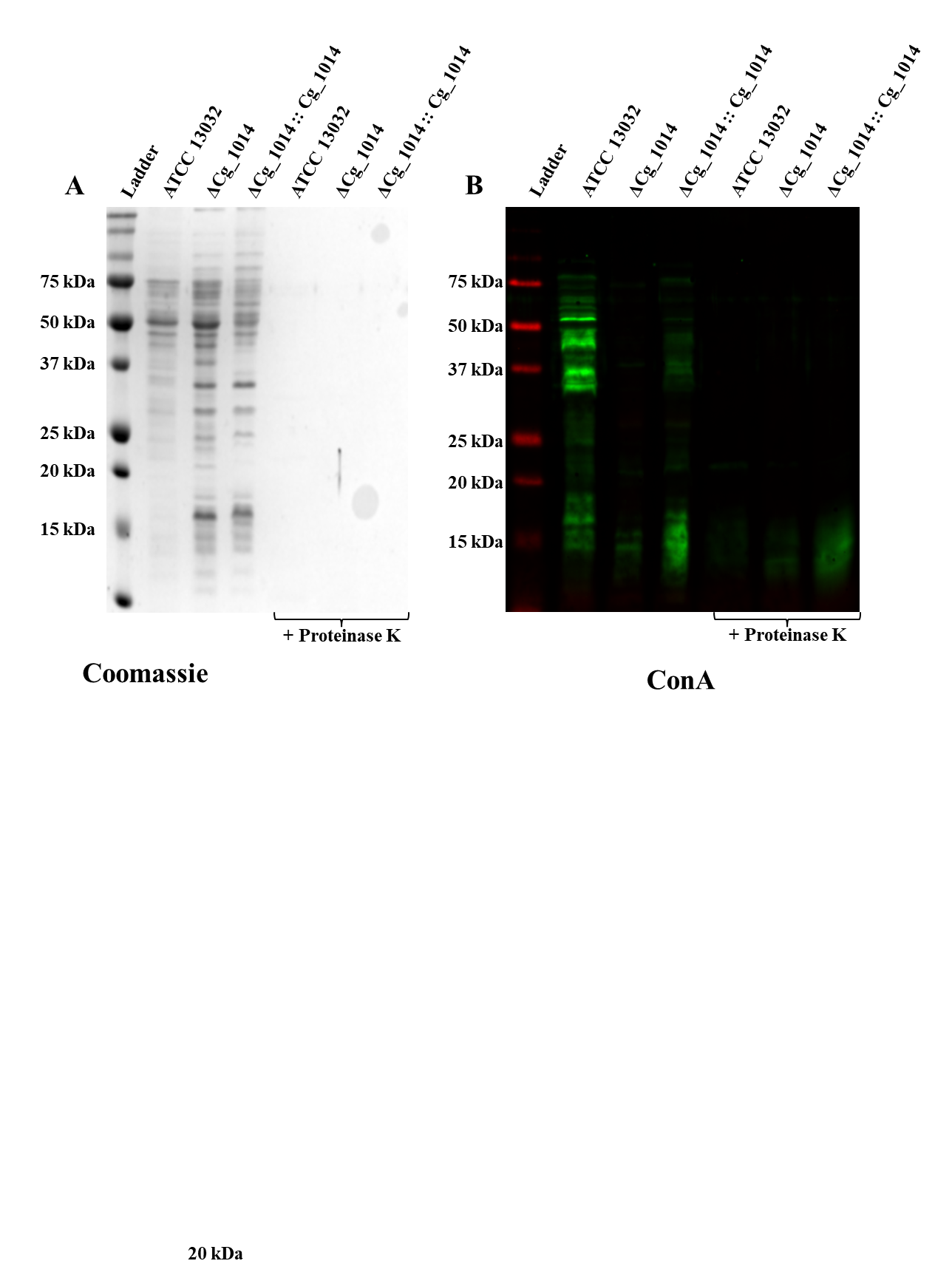
**

**Figure S3: Coomassie stained 15% SDS-PAGE (A) and ConA-FITC (green) lectin blot (B) of *C. glutamicum* ATCC 13032, ΔCg_1014, and ΔCg_1014:Cg_1014 membrane fractions following digestion with proteinase K. Coomassie stained gel (A) shows membrane fractions before (Lanes 2 – 4) and after proteinase K digestion (Lanes 5 – 7).** Digestion of proteins in each membrane fraction is evident by lack of Coomassie strained bands in proteinase K treated samples (A). ConA reactive smears in lectin blot (B) of membrane fractions from *C. glutamicum* ATCC 13032, ΔCg_1014, and complemented strains are attributed to the presence of LAM in the samples. Distinct bands that remain in ATCC 13032 and ΔCg_1014:Cg_1014 membrane fractions following proteinase K digest (Lanes 5- 7) are attributed to protease resistant mannoproteins as POM is known to confer proteolytic resistance. Molecular weight standards are the Bio-Rad All Blue ladder.

**
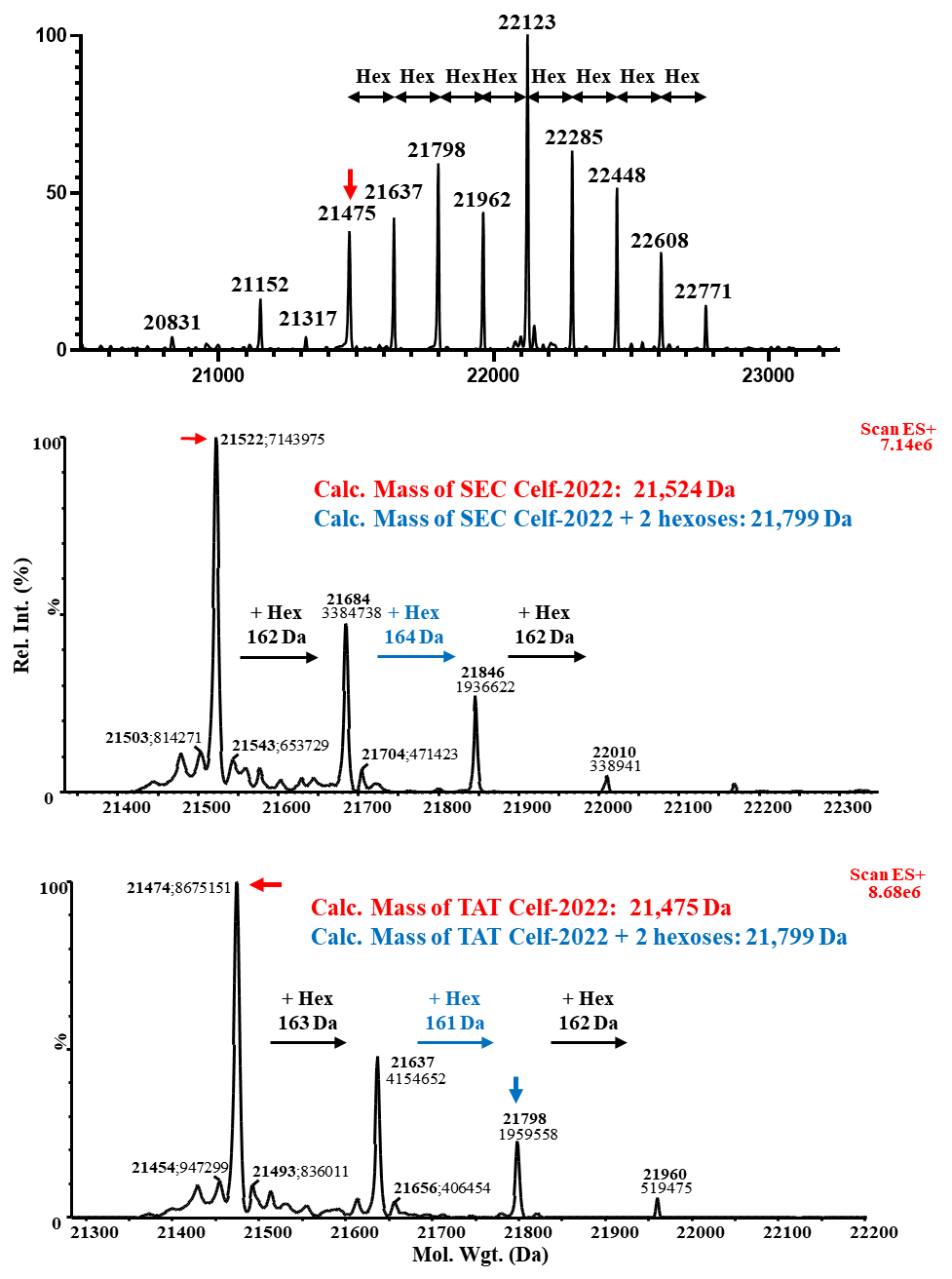
**

**Figure S4: Hexose modifications by intact mass LC-MS analysis of Celf_2022 expressed in *C. glutamicum* ATCC 13032 using its native and unknown signal peptide (top panel), the SEC signal peptide of Celf_1230 (middle panel), and the TAT signal peptide of Celf_3184 (bottom panel).** All proteins were recovered from the spent culture media. The calculated mass (with signal peptide removed and no hexose modifications) of Celf_2022 is 21,475 Da except in the case of the SEC signal peptide fused Celf_2022 where a single amino acid mutation in the mature protein caused the calculated mass to increase to 21,524 Da. When expressed with the native and unknown secretion signal, Celf_2022 is exported and modified by up to 8 hexoses. Replacement of this signal peptide by either the SEC or TAT signal peptides of Celf_1230 or Celf_3184 reduces the maximum number of hexose modifications to 4 or 3, respectively.

**
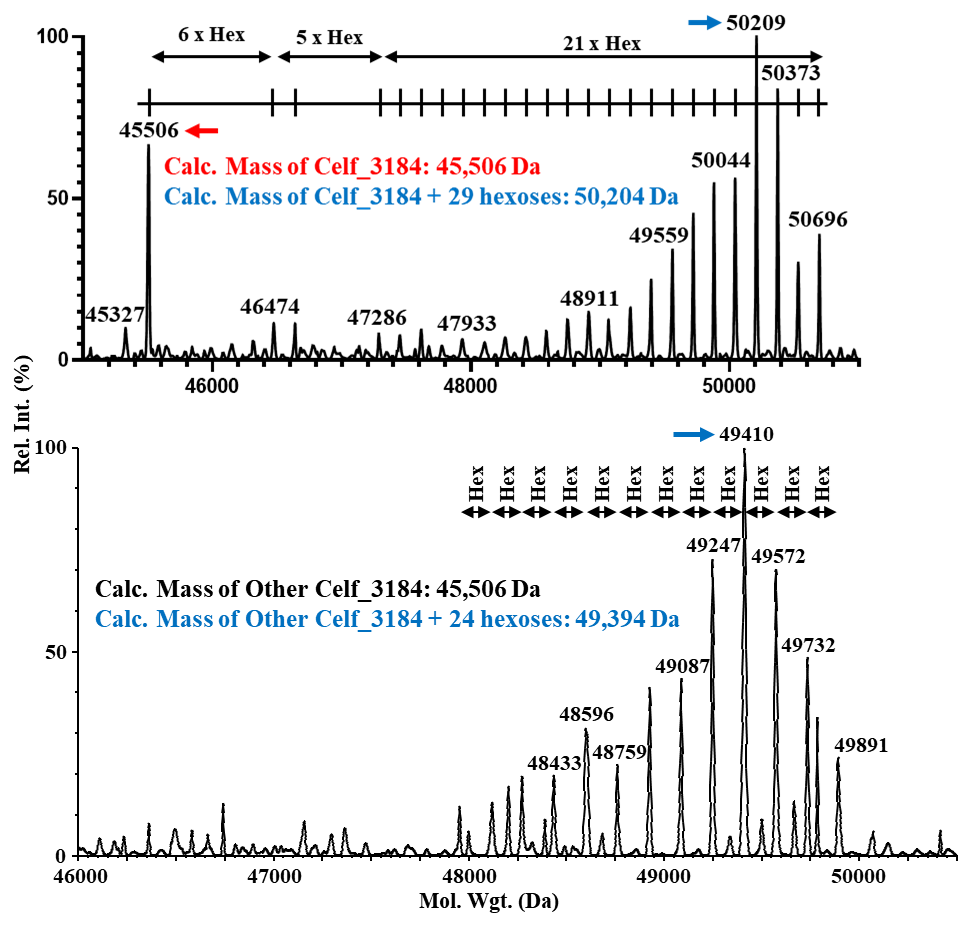
**

**Figure S5: Hexose modifications by intact mass LC-MS analysis of Celf_3184 expressed in *C. glutamicum* ATCC 13032 using its native TAT signal peptide (top panel) and unknown signal peptide of Celf_2022 (bottom panel).** Both proteins were recovered from the spent culture media. The calculated mass (with signal peptide removed and no hexose modifications) of Celf_3184 is 45,506 Da. The native TAT secretion signal of Celf_3184 results in 14 – 32 hexose modifications. Replacement by the unknown signal peptide of Celf_2022 results in a narrower (16 – 26) profile of hexose modifications with no detectable unmodified protein.

**
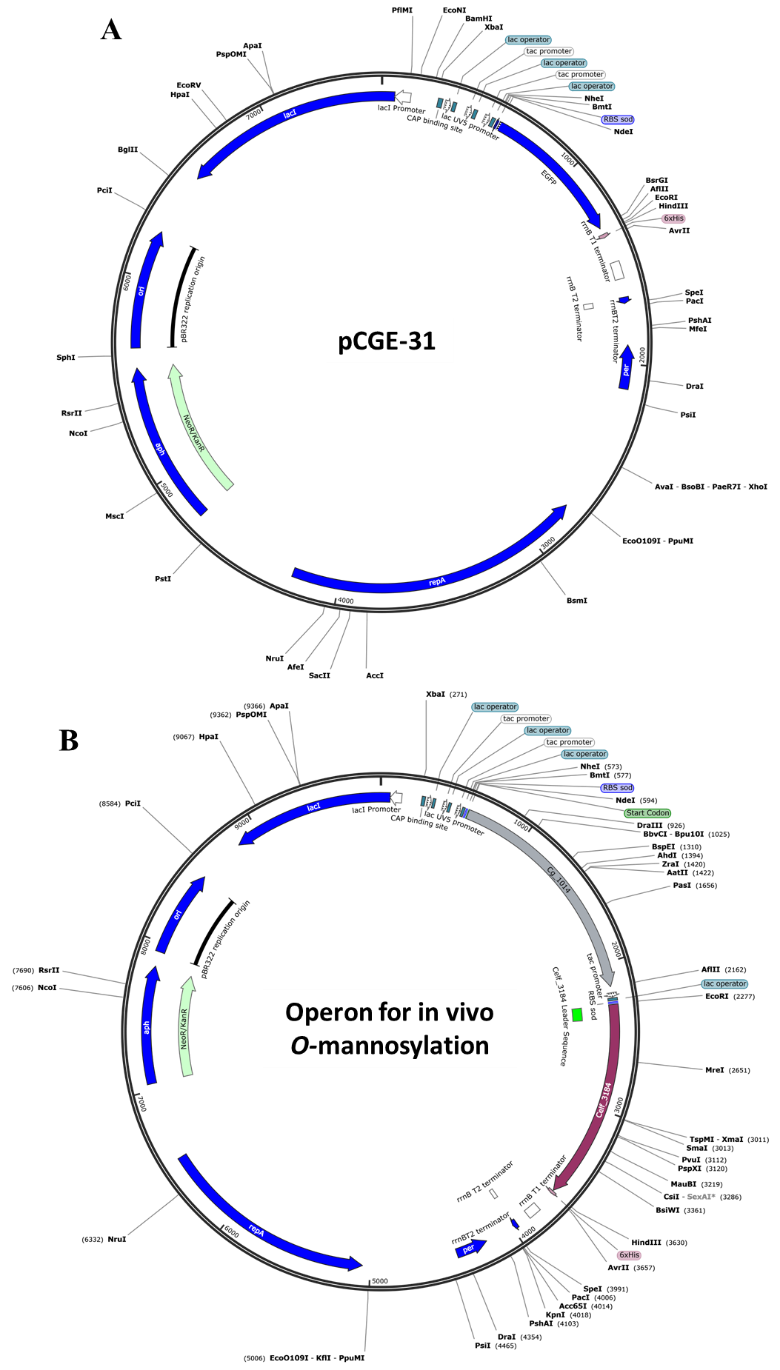
**

**Figure S6: Plasmid maps of pCGE-31 used for recombinant expression of GT-39s (A) and *O*-mannosylation operons used for co-expression of GT-39s with target actinobacterial mannoproteins (B).** Features of pCGE-31 *E. coli*/*C. glutamicum* shuttle vector (A). The pCGE-31 vector originates from pTGR-5 and received the triple promoter system from pCW via restriction cloning. Actinobacterial GT-39 genes amplified from genomic DNA replaced eGFP using NdeI and HindIII restriction sites. Features of the *O*-mannosylation operon constructs (B). A synthetic secondary ORF containing the Celf_3184 gene was added via restriction cloning (NdeI – AvrII) under the control of Ptac and also utilizing the sod RBS.

**Table S1: Global sequence similarity of actinobacterial GT-39s compared to *S. cerevisiae* PMT1.** Global sequence similarity was determined using EMBOSS Needle pairwise sequence alignment.

|  | ***S. cerevisiae* (PMT1)** | ***M. tuberculosis*** | ***M. smegmatis*** | ***C. glutamicum*** | ***C. fimi*** | ***C. flavigena*** |
| --- | --- | --- | --- | --- | --- | --- |
| ***S. cerevisiae* (PMT1)** | **100.0%** | 25.7% | 25.1% | 26.0% | 25.4% | 23.2% |
| ***M. tuberculosis*** | 25.7% | **100.0%** | 83.9% | 55.9% | 46% | 46.9% |
| ***M. smegmatis*** | 25.1% | 83.9% | **100.0%** | 57.7% | 46% | 48.7% |
| ***C. glutamicum*** | 26.0% | 55.9% | 57.7% | **100.0%** | 42% | 40.5% |
| ***C. fimi*** | 25.4% | 46.0% | 46% | 42.0% | **100.0%** | 50.5% |
| ***C. flavigena*** | 23.2% | 46.9% | 48.7% | 40.5% | 50.5% | **100.0%** |

**Table S2: Selected primers used in this study.**

| ***Target Amplicon*** | ***5’*** | ***3’*** |
| --- | --- | --- |
| ***Cg_1014*** | 5'-TTG ATT CAT ATG GTG AGC CAA GCC CTA CCT GTT CG-3’ | 5'-TTG ATT CTT AAG TTA GCG CCA GCT TGG GAA CCA CAT CAA GG-3’ |
| ***Celf_3080*** | 5'-TTG ATT CAT ATG GTG CCG CCC ACG CGA GAC GAC-3’ | 5'-TTG ATT CTT AAG TCA GAT CCA GCT CGT CAG CCA CAT GTG GCT GTG C-3’ |
| ***Cfla_0843*** | 5'-TTG ATT CAT ATG GTG CCG ACC GAC GGA GAC GAC ACC GAG-3’ | 5'-TTG ATT CTT AAG TCA GAT CCA GGT CGG CAG CCA CAT CCG GAT GTG C-3’ |
| ***pCW triple promoter*** | 5'-ATT AGT CTA GAT AAT GTG AGT TAG CTC ACT CAT TAG G-3’ | 5'-ATT AGG CTA GCA AAT TGT TAT CCG CTC ACA ATT CCA-3’ |
| ***Cg_1014 upstream*** | 5'-GGG GAT CCT TCT TCG GTT GCG GTA ATT TGC TCT GGC T-3’ | 5'-AGC CAT CTC TCA CTC GGT TGA TTG TAG AGC CTT GGC-3’ |
| ***Cg_1014 downstream*** | 5'-TAG ATC GCC CTC CCC TTT TAC CGC ACC AGG TGA CC-3’ | 5'-CGA CTC TAG AGT CGA TGT CAT GAA CCA CTG GCT CGA C-3’ |
| ***Cg_1014 deletion*** | 5'-GGG GAT CCT TCT TCG GTT GCG GTA ATT TGC TCT GGC T-3’ | 5'-CGA CTC TAG AGT CGA TGT CAT GAA CCA CTG GCT CGA C-3’ |
| ***C. glutamicum MBP*** | 5'-TTG AAG CGT CTT ACT CGC ATC GCA TCC ATC-3’ | 5'-TTA GCC CCA GTT GGA TTC CTT CTC AGC AG-3’ |
| ***celf_3184*** | 5'-GGG GTA TTC CAT ATG TCC ACC CGC AGA ACC GCC GCA GCG-3’ | 5'-GGG GAA TTC TCA CCA CCT GGC GTT GCG CGC CAT C-3’ |
| ***pCGE-31 MCS*** | 5'-GCA TGA TAT GGA TCC ATA TAT GCG GCC GCA TAT TCT AGA-3’ | 5'-GCG CTA CGG CGT TTC ACT TCT GAG-3’ |
| ***SEC – Celf_2022 N-term*** | 5'-ATT AGC ATA TGG TGG CCC GAC CCT TCC G-3’ | 5'-ACG AGT CAG CGC GGC CGC GG-3’ |
| ***SEC – Celf_2022 C-term*** | 5'-CGG CCG CGC TGA CTC GTG ACG ACA CTC CTG TGG-3’ | 5'-ATT AGG AAT TCT TAT CAA TGG TGA TGG TGA TGG TGC TGG GT-3’ |
| ***TAT – Celf_2022 N-term*** | 5'-CAT ATG TCC ACC CGC AGA ACC GCC-3’ | 5'-CAC GAG TCA GCG CCT GCG CGG CGG TG-3’ |
| ***TAT – Celf_2022 C-term*** | 5'-GCG CAG GCG CTG ACT CGT GAC GAC ACT CCT-3’ | 5'-AAG CTT AGA ATT CTT ATC AAT GGT GAT GGT GAT GGT GC-3’ |
| ***“Other” – Celf_1230 N-term*** | 5'-ATT AGC ATA TGT CCA GCA AGC GCG AAC GTG AG-3’ | 5'-GCC GCC GTG TGC CAC AGC TGC ACC GAT ACC C-3’ |
| ***“Other” – Celf_1230 C-term*** | 5'-GCT GTG GCA CAC GGC GGC CCC CC-3’ | 5'-ATT AGA AGC TTG CTG CGC GGG C-3’ |
| ***“Other” – Celf_3184 N-term*** | 5'-ATT AGC ATA TGT CCA GCA AGC GCG AAC GTG AG-3’ | 5'-AGC CGG GAG CCG CCA CAG CTG CAC CGA TAC CC-3’ |
| ***“Other” – Celf_3184 C-term*** | 5'-GCT GTG GCG GCT CCC GGC TGC-3’ | 5'-ATT AGA AGC TTC CAC CTG GCG TTG CG-3’ |
